# Bone metastasis of malignant thymomas associated with peripheral T-cell lymphocytosis

**DOI:** 10.1186/s12893-016-0171-0

**Published:** 2016-08-19

**Authors:** Luo Zhao, Xi Zhou, Zheng Li, Yong Liu

**Affiliations:** Department of Orthopaedic Surgery, Peking Union Medical College Hospital, Beijing, China

**Keywords:** T-cell lymphocytosis, Malignant thymoma

## Abstract

**Background:**

Malignant thymomas are rarely associated with bone metastasis and T-cell lymphocytosis.

**Case presentation:**

A 47–year-old female patient was admitted to our hospital for ptosis. A diagnosis of malignant thymoma was made based on the thymectomy and pathological result. Peripheral T-cell lymphocytosis and bone metastasis were found later. T-cell lymphocytosis was relived after surgical and radiation therapy to the metastasis.

**Conclusion:**

Peripheral T-cell lymphocytosis is a rare paraneoplastic phenomenon associated with thymomas. This report is the first to describe an invasive thymoma with late bone metastasis accompanied with T-cell lymphocytosis. We should be aware of peripheral T-cell lymphocytosis in thymomas and it may contribute to a better understanding of the complex physiology and pathogenesis of thymoma.

## Background

Thymomas are rare anterior mediastinal tumors, which originate from the thymic epithelial cells. According to the pathologic classification developed by the World Health Organization, thymomas are classified into five groups (type A, AB, B1, B2 and B3) by predominate cell type. Thymomas are usually slow growing with more local recurrence rather than metastasis. Sporadic cases with late distant metastasis, including lungs, pleura, liver, thyroid and bones, have been reported [1].

The thymus gland is responsible for the maturation of T-lymphocyte and some other immunologic function. Thus, the neoplastic thymoma is associated with an array of “paraneoplastic” syndromes such as myasthenia gravis, pure cell aplasia, hypogammagloulinemia. However, T-cell lymphocytosis in peripheral blood is an extremely rare phenomeon.

We report a 47-year-old female patient with a previous history of myasthenia gravis (MG) and malignant thymoma, who developed thoracic vertebrae and paravertebrae metastasis associated T-cell lymphocytosis.

## Case presentation

A 47–year-old female patient was admitted to our hospital in 2007 for ptosis. Laboratory tests showed higher percentage of the lymphocytes (77.5 %) with 4.45 × 10^9^/L leukocytes and 3.45 × 10^9^/L lymphocytes. A chest computed tomography scan showed an 11 cm × 9 cm × 9 cm irregular solid mass in the anterior mediastinum, with infiltration of the diaphragm and left chest wall. She was diagnosed with MG and a mediastinal mass. Thymectomy detected a mediastinal mass infiltrating the pericardium and left pleura and penetrating the left lung. In addition, second mass was found adherent to the left diaphragm. Both tumors were resected and histologically classified as malignant thymoma (type AB) with infiltration of chest wall, diaphragm, pericardium, left pleura and lung. Regional nodules were resected and showed lymphadenitis. The patient received ADOC chemotherapy (Cyclophosphamide, Doxorubicin, Cisplatin, Vincristine) and radiation therapy after surgery and followed up with a relief of MG.

The patient followed up with chest CT every year and local recurrence in the left pleura was found in April, 2010. Unfortunately, the patient was found to have peripheral T-cell lymphocytosis in 2014. Peripheral blood analysis showed mild lymphocytosis (5.3 × 10^9^/L), with Lymphocytes representing 60.7 % of the peripheral blood leukocytes. Flow cytometric analysis revealed that most of the lymphocytes were mature CD3+ CD5 + CD7+ T cells. Moreover, bone marrow aspiration and biopsy showed normal cellular counts and percentage.

**Fig. 1:**
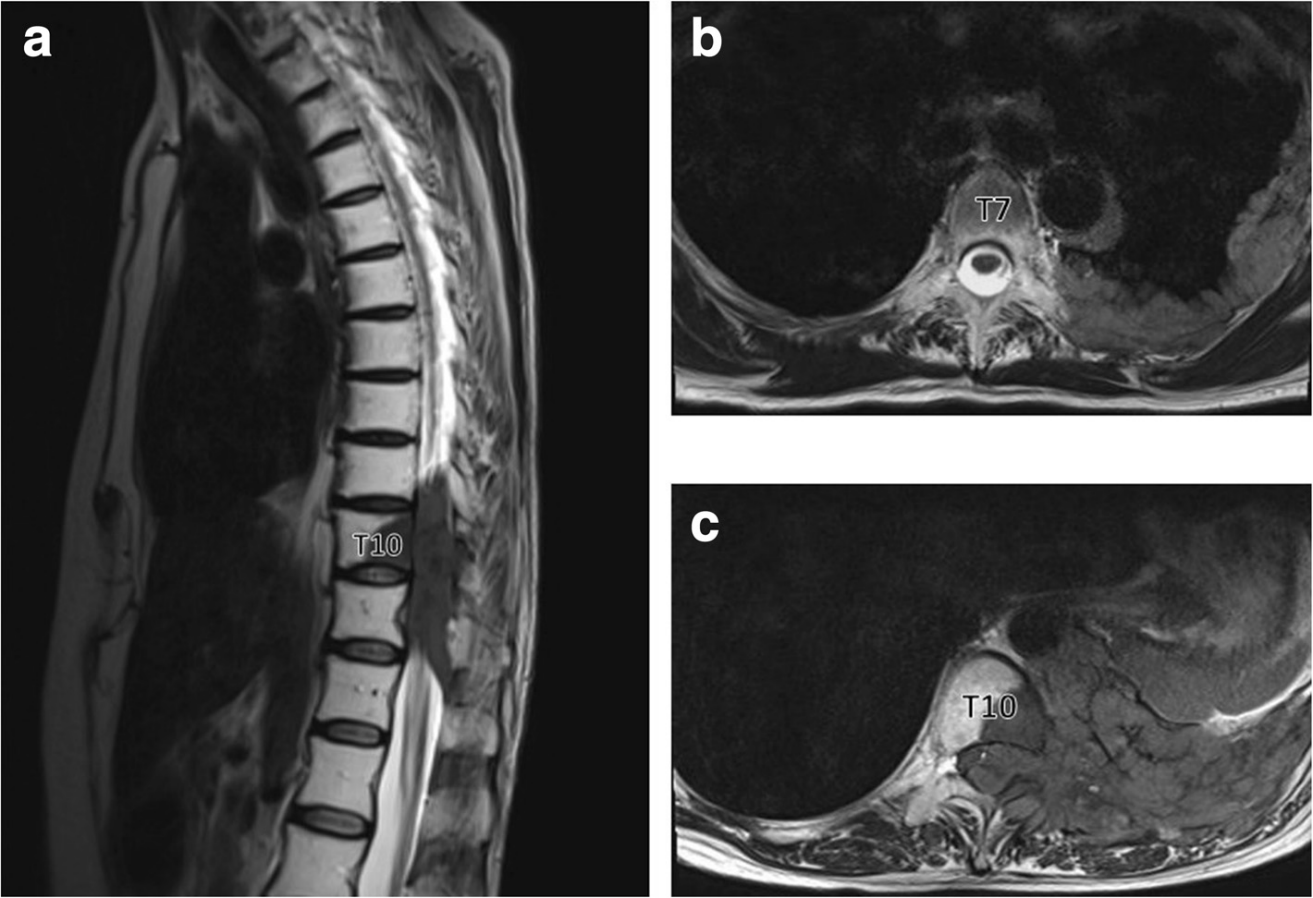
Preoperative T2-weighted magnetic resonance images of thoracic spine. **a**. sagittal image revealed a paravertebral mass between T9 and T12 level. **b**. coronal image of T7 vertebrae showed pleural mass. **c**. coronal image of T10 vertebrae showed spinal compression

**Fig. 2:**
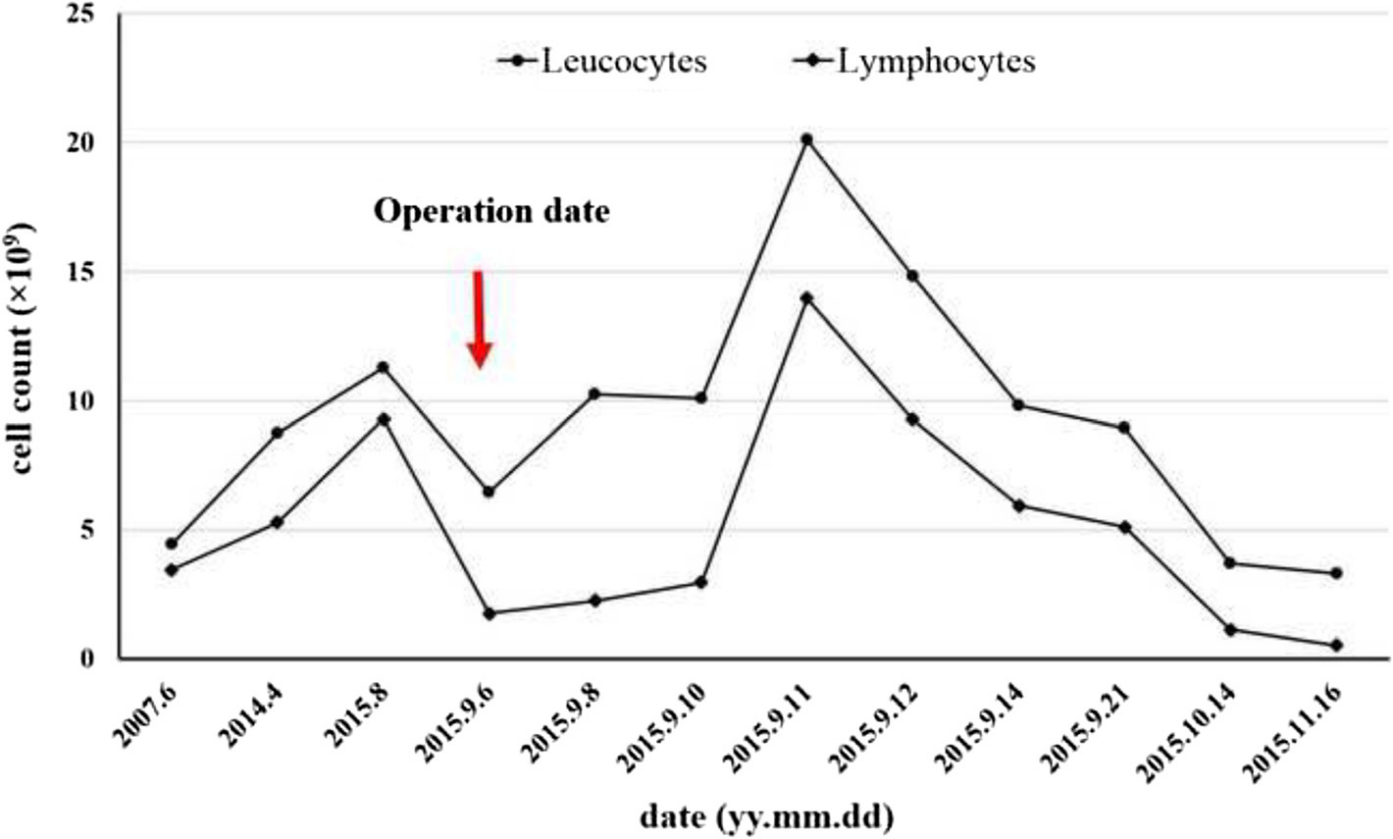
Variation of peripheral leucocytes and lymphocyte count

The patient developed back pain and progressive lower extremity weakness in August 2015. She also complained of hypesthesia of lower limbs with urine incontinence. The thoracic magnetic resonance imaging (MRI) showed a paravertebral mass between T9 to T12 level with spinal cord compression (Fig. 1). Laboratory tests showed mild leukocytosis (11.29 × 10^9^/L) with lymphocytosis (9.29 × 10^9^/L). We performed posterior decompression, tumor resection with bone cement reconstruction and internal fixation emergently. Immediately after the operation, the patient’s peripheral lymphocyte count decreased markedly from 5.61 × 10^9^/L preoperatively to 1.77 × 10^9^/L. However, the lymphocyte count increased gradually to 13.97 × 10^9^/L 4 days after surgery, and remained elevated till discharge. One month later, she received radiation therapy again and the lymphocytosis resolved during the therapy (Fig. 2). The pathology reported malignant thymoma (type B2, locally B3), and her muscle strength and urinary function recovered gradually.

## Discussion

Thymomas are one of the most common tumors in the anterior mediastinum. Malignant thymomas are usually determined by the invasiveness into nearby tissues or distant metastasis. The recurrence of thymomas is rare, and distant metastasis is reported by only a few cases [1]. In the present study, the patient was diagnosed with malignant thymoma with diaphragm and pleural infiltration at the onset. Interestingly the pathology classified the thymoma into type AB from the onset but progressed into type B2/B3 in the bone metastasis 8 years later. We hypothesized that the biological behavior of thymomas might have changed or progressed during time.

On rare occasions peripheral T-cell lymphocytosis is associated with malignant thymomas. Only a few cases have been reported, but the mechanism has not been well described until now. For example, Pedraza et al. reported the first case in 1977 [2], and Barton reviewed seven similar cases in 1997 [3]. A few more cases were reported after then (Table 1). It seems that females were more likely to suffer from this situation than males, which may help us to clarify the mechanism. In all these cases, T-cell lymphocytosis was asscociated with aggressive thymomas, and most of them were relived after surgery or chemo/radiation therapy. On these conditions, lymphoproliferative diseases should be first excluded, although it is more rarely coexist with thymomas. In our case, the flow cytometric study showed a polyclonal T-cell population. The bone marrow examination also excluded bone marrow lymphocytosis as well as lymphocytic leukemia. Therefore, we concluded that the T-cell lymphocytosis originated from peripheral.

**Table 1:** Case review

Author, year	Presentation	Invasive or metastasis	Peripheral lymphocytes	Therapy	Outcome
Pedraza, 1977 [2]	69y/male	Left pleura	15,000 leukocytes with 70 % lymphocytes	Surgery/radiotherapy	Resolved
Griffin et al., 1978 [5]	64y/female	N/A	25,000 leukocytes with 52 % lymphocytes	Biopsy/chemotherapy	Resolved
Shachor et al., 1988 [6]	37y/female	Heart/hilum/lung/pleura/chest wall	28,000 leukocytes with 87 % lymphocytes	Biopsy/chemotherapy/radiotherapy	Resolved
Medeiros et al., 1993 [4]	66y/male	Lung	20,000 leukocytes with 80 % lymphocytes	Biopsy/chemotherapy/radiotherapy	Not resolved
Smith et al., 1994 [7]	80y/female	Soft tissue of the neck/ left brachiocephalic vein	11,700 leukocytes with 59 % lymphocytes	Surgery/radiotherapy	Resolved
Smith et al., 1994 [7]	55y/female	Left pleura	16,300 leukocytes with 52 % lymphocytes	Surgery/chemotherapy/radiotherapy	Resolved
Barton, 1997 [3]	35y/female	Right pleura	20,100 leukocytes with 57 % lymphocytes	Surgery/radiotherapy	Resolved
Otton SH et al.2000 [8]	71y/male	N/A	6500 lymphocytes	Steroids & azathioprine	Not resolved
Morales et al., 2007 [9]	36y/male	Lung/right pleura/chest wall	13,800 leukocytes with 67 % lymphocytes	Surgery/chemotherapy/radiotherapy	Not resolved
Chen HK et al., 2009 [10]	62y/female	Lung	18,000 leukocytes with 77.4 % lymphocytes	Surgery	Resolved
Puljiz Z et al., 2013 [1]	76y/female	Lung/liver	34,400 leukocytes with 57 % lymphocytes	Surgery/chemotherapy/radiotherapy	Partially resolved

In our case, the percentage of lymphocyte was higher than normal in 2007, and T-cell lymphocytosis was diagnosed in 2013. No therapy was conducted until the patient developed lower limb weakness. Interestingly, T-cell lymphocytosis got relived immediately after the removal of the vertebral tumor. However the lymphocyte count elevated 4 days later. The radiation therapy was done and the lymphocyte counted decreased to normal again. Therefore, we concluded that T-cell lymphocytosis was associated with malignant thymoma in this case. Previous cases showed that most T-cell lymphocytosis was relived after tumor resection or chemotherapy/irradiation, indicating that lymphocytosis might be one of the paraneoplastic phenomena of thymoma. In addition, T-cell lymphocytosis may be a prediction whether the thymoma is sensitive to the therapy.

However, the mechanism has not been fully elucidated. In most cases T-cell lymphocytosis quickly resolved after surgical resection and/or chemo/radiotherapy. Earlier studies indicated a spillover from the lymphocyte-rich thymoma into the peripheral blood [3]. However, later studies found differences between the immunophenotype of the cells in thymoma and the peripheral T-cells. Given that the immunophenotypes of circulating lymphocytes were polyclonal mature T cells, these cells were postulated to derive from the spillage of intratumoral lymphocytes into the circulation or a reactive proliferation of peripheral lymphocytes, which was related to deregulated, thymoma-associated immunoresponse. Medeiros suggested there was an immunoregulatory disorder mediated by thymic hormone released by the tumor. Most authors now agree that there may be a physiologic imbalances with malignant thymic cells, which lead to immune disorders and finally to impaired proliferation, maturation, and migration of T-cells [4].

## Conclusion

Peripheral T-cell lymphocytosis is a rare paraneoplastic phenomenon associated with thymomas. We propose that female sex, lymphocyte-rich thymomas that behaved aggressively and invasively may be the risk factors in the majority of cases. As far as we know, our report is the first to describe an invasive thymoma with late bone metastasis accompanied with T-cell lymphocytosis. Clinicians should be aware of peripheral T-cell lymphocytosis in thymomas, although it is a rare manifestation of thymomas. It contributes to a better understanding of the complex physiology and pathogenesis of thymoma. Moreover, further studies are needed to investigate the relationship between T-cell lymphocytosis and thymoma.

## Abbreviations

ADOC, cyclophosphamide, doxorubicin, cisplatin, vincristine; MG, myasthenia gravis; MRI, magnetic resonance imaging
